# Effects of Dental Implants and Nutrition on Elderly Edentulous Subjects: Protocol for a Factorial Randomized Clinical Trial

**DOI:** 10.3389/fnut.2022.930023

**Published:** 2022-06-27

**Authors:** Shu-Jiao Qian, Beilei Liu, Junyu Shi, Xiao Zhang, Ke Deng, Jie Shen, Yang Tao, Shichong Qiao, Hong-Chang Lai, Changzheng Yuan, Maurizio S. Tonetti

**Affiliations:** ^1^Shanghai PerioImplant Innovation Center, Department of Oral and Maxillofacial Implantology, National Clinical Research Center of Stomatology, Ninth People's Hospital, College of Medicine, Shanghai Jiao Tong University, Shanghai, China; ^2^School of Public Health, Zhejiang University School of Medicine, Hangzhou, China; ^3^Department of Nutrition, Harvard T. H. Chan School of Public Health, Boston, MA, United States; ^4^European Research Group on Periodontology, Genova, Italy

**Keywords:** tooth loss, masticatory function and nutrition, diet, nutrition—clinical, healthy aging, randomized control trial (RCT)

## Abstract

**Background:**

Loss of masticatory function consequent to tooth loss has been associated with changes in food choices and insufficient nutritional intake. To date, interventions based on dental prostheses alone did not significantly improve nutrient intake. Pilot studies have shown positive impacts of interventions combining implant-supported fixed dental prosthesis with brief dietary advice. The relative contribution and the potential synergy of the components of such interventions need to be determined as it has major public health implications for the community-dwelling aging population that continues to disproportionately suffer from tooth loss and its consequences.

**Objective:**

To assess the effect of rehabilitation of masticatory function with fixed implant supported dentures and nutrition education in older subjects with terminal dentition (stage IV periodontitis) or full edentulism.

**Methods:**

A 2 × 2 factorial randomized controlled trial with 16-month follow-up of eligible adults (≥60 years) with loss of masticatory function consequent to full arch edentulism or terminal dentition (*n* = 120) will be conducted to test whether the rehabilitation of masticatory function with fixed implant supported dentures, nutrition education and/or their combination improves intake of fresh fruits and vegetables for aging subjects. The study has been designed to detect changes in fresh fruits and fresh vegetables intake at 4 months using the 24-h dietary recall method. Changes in protein as percentage of total energy, nutritional biomarkers, plasma metabolomics, oral and gut microbiome, quality of life and masticatory function will also be assessed.

**Discussion:**

We hypothesize that receiving rehabilitation of masticatory function with fixed implant dentures together with nutrition education is the most effective intervention for improving nutrient intake in aging community-dwelling subjects with extensive tooth loss. The results of this study will assist in designing better treatment regimens, guide medical care for individual subjects, and inform public health and policy.

**Clinical Trials Registration:**

NCT05334407.

## Introduction

Over the course of life unmanaged caries and periodontitis, the most common diseases of mankind, lead to tooth loss and associated loss of quality of life and eventually compromised masticatory function. Older adults and aging subjects may be disproportionately affected (1). At the end of the disease spectrum, subjects with complete tooth loss (edentulism) or presence of only few remaining teeth that do not enable adequate chewing function show changes in their food choices and seem to prefer softer diets with higher carbohydrates and fat and less fresh fruits and vegetables (2, 3).

Accumulating evidence points to the presence of an association between changes in dietary behavior consequent to tooth loss and insufficient nutrition intake (4–6). A recent systematic review indicated that subjects lacking a functional dentition had a 21% increased likelihood of being at risk of malnutrition or being malnourished (7). Such impaired nutrition may have long term effects on muscle strength and physical decline and be detrimental to general health (8, 9). Indeed, the recent Global Burden of Disease study of dietary risk factors identifies 15 important disease associated exposures. Their analysis shows that 5 of the health associated exposures: consumption of fruit, vegetables, whole grains, nuts, and fiber require a good level of mastication (10).

A recent systematic review has addressed the efficacy of tooth replacement with dental prostheses and identified clear benefits in terms of restoration of masticatory function (11). Among fully edentulous subjects, greater benefits have been observed with dental implant retained prostheses with respect to conventional dentures.

While the physiology of mastication is an essential component of alimentation and contributes to the broader process of nutrition, recent research has focused on the nutritional benefits of tooth replacement to better focus the relevance of oral health on general health. Several studies have tried to improve the nutrient intake among edentulous individuals with various types of dentures. However, this goal has been elusive for interventions based on either complete dentures or implant-retained overdentures, given the functional limitation on these prostheses and perhaps the lack of concomitant dietary intervention (12–15). A small-scale case series has shown that implant-supported fixed prosthesis resulted in more efficient mastication and improved nutrient intake compared with conventional and implant-based removable dentures in partial edentulism (16).

Within dentistry, the long-held assumption that restoration of masticatory function alone—i.e., without dietary re-education intervention—brings nutritional benefits is being questioned. Sparse evidence points to the positive impact of nutrition counseling on the dietary intake of edentulous subjects receiving dental prostheses: brief dietary advice has been advocated to help patients take full advantage of the enhanced masticatory function to improve their diet (17, 18). Ellis et al. further showed that the impact of dietary advice on patient's satisfaction with dentures and oral health-related quality of life depends on the nature of the prosthesis (19). A recent systematic review on the impact of oral rehabilitation coupled with dietary advice on nutritional status has indicated that in most studies the dietary interventions were not theory based and poorly described (20). Not unexpectedly, the meta-analysis found only a trend toward significant changes in fruit and vegetable consumption and marked heterogeneity among the included pilot case series. No trial has been performed to assess the benefit of dietary advice alone or the combined effect of re-establishment of masticatory function with an implant-supported fixed prosthesis and dietary advice in edentulous elderly subjects. Understanding the relative contribution of restoration of masticatory function and nutrition education is critical to design effective interventions and improve public policy related to nutrition and prevention of physical decline in aging populations.

Based on the current equipoise about the relative contribution of dental and dietary interventions and the clinical and public health relevance of defining appropriate interventions to improve nutrition of older adults with extensive tooth loss, this protocol describes a 2 × 2 factorial clinical trial to assess the effect of rehabilitation of masticatory function with fixed implant supported dentures and/or brief nutrition education on the dietary intake and nutrition in older subjects with terminal dentition (stage IV periodontitis) or full edentulism. The clinical trial is being implemented. The effectiveness of the intervention will be validated during the trial. Results will identify the relative importance and optimal sequence of dental and dietary interventions, providing critical information with major implications for caring of individual subjects and for public health and policy. The results of the clinical trial will be available in 2 years.

## Methods and Materials

### Study Design

This protocol has been prepared according to the SPIRIT guideline for clinical trial protocols (21).

#### Study Overview

**Figure 1:**
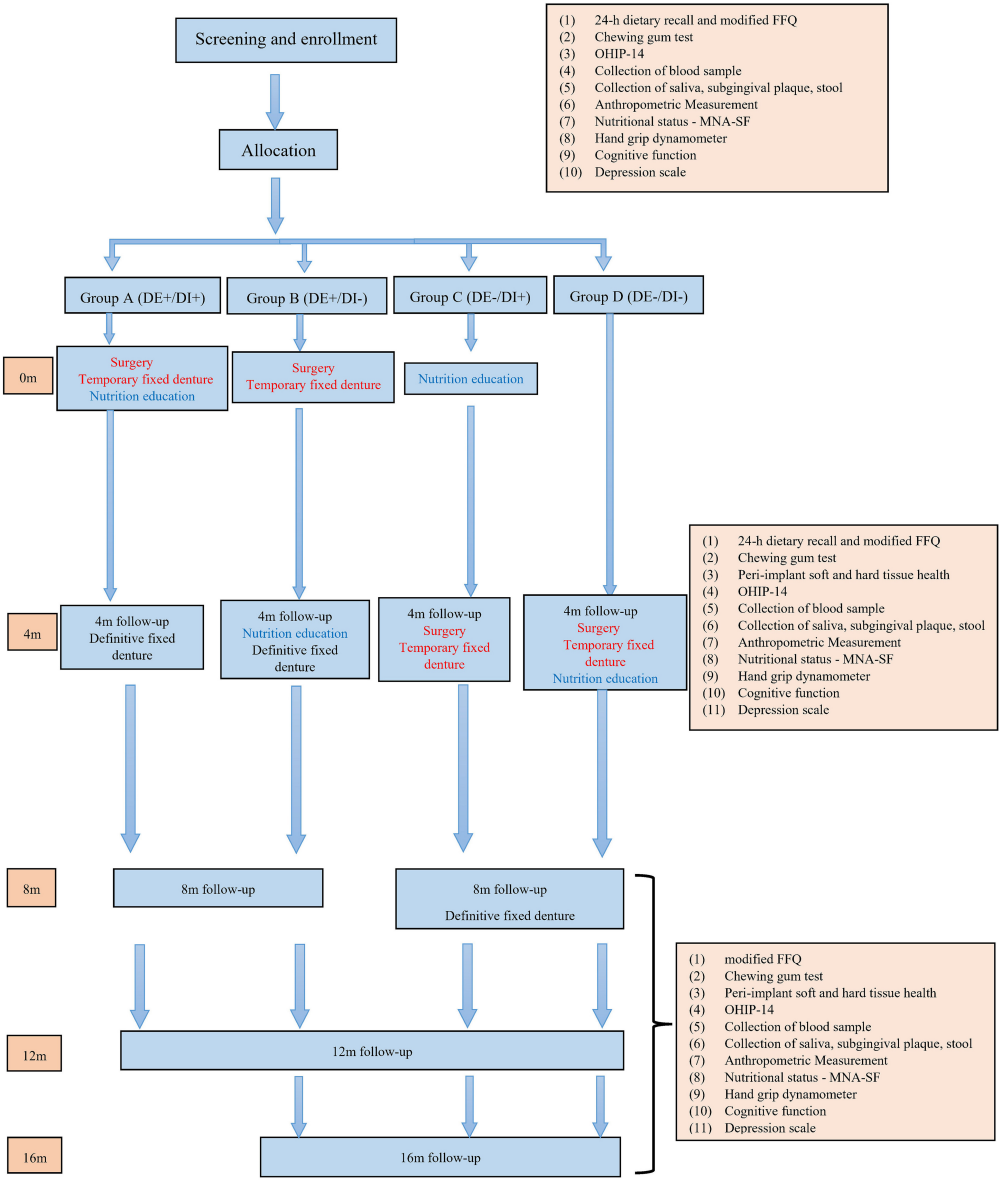
Study flow chart.

The study is designed as a factorial randomized controlled clinical trial testing the benefits of dental and/or dietary interventions on changes in fresh fruits and fresh vegetables intake at 4 months (Table 1). Group A (DE+/DI+) will receive the full treatment regimen. Group B (DE+/DI–) will receive implant-supported fixed prosthesis at first and nutrition education after a 4-month waiting period. Group C (DE–/DI+) will receive the nutrition education at first and implant treatment after a 4-month waiting period. Group D (DE–/DI–) will receive the same treatment as Group A after a 4-month waiting period (Figure 1). The waiting period is equal to the current waiting list in the department. Subjects will be followed for an additional 12-month period. All procedures will follow the principles of the Declaration of Helsinki on experimentation involving human subjects, all subjects will provide written informed consent. The trial has been approved by the Institutional Review Board of the Shanghai Ninth People's Hospital, Shanghai Jiao Tong University School of Medicine (Approval No. SH9H-2021-T321-3) and is registered in ClinicalTrials.gov (NCT05334407). The trial will be independently monitored by the office of clinical research of the Shanghai Ninth People's Hospital. Any modifications to the protocol with impact on the conduct of the study will require a formal amendment to the protocol. The SPIRIT summary of the trial procedures is illustrated in Supplementary Table 1.

**Table 1:** Overview of the schedule of enrolment, interventions, and assessments.

	**Study period**							
	**Enrolment**	**Allocation**	**Post-allocation**					**Close-out**
**Timepoint**	**−7 ±7 d**	**0**	**−0 ±7 d**	**4 m ±14 d**	**8 m ±14 d**	**12 m ±14 d**	**16 m ±14 d**	
Intervention^*^			**DE**	**DI**	**DE**	**DI**		
**Enrolment**								
Eligibility screen	X							
Informed consent	X							
Demographics	X							
Medical history	X							
Concomitant medications	X							
Allocation		X						
**Interventions**								
[Group A:DE+/DI+]			X	X				
[Group B:DE+/DI–]			X			X		
[Group C:DE–/DI+]				X	X			
[Group D:DE–/DI–]					X	X		
**Assessments**								
*24-h dietary recall*	X			X				
*food-frequency questionnaire*	X			X	X	X	X	
*Anthropometric measurement*	X			X	X	X	X	
*Masticatory function*	X			X	X	X	X	
*Peri-implant soft tissue condition*				X	X	X	X	
*OHIP-14*	X			X	X	X	X	
*Blood sample*	X			X	X	X	X	
*Saliva sample*	X			X	X	X	X	
*Subgingival plaque sample*	X			X	X	X	X	
*Stool sample*	X			X				
*Nutritional status*	X			X	X	X	X	
*Muscle strength*	X			X	X	X	X	
*Cognitive function*	X			X	X	X	X	
*Depressive symptoms*	X			X	X	X	X	

* *Interventions included DE (dental intervention) and DI (Dietary Intervention)*.

#### Recruitment

Older subjects (≥60 years of age) with full arch edentulism or terminal dentition seeking care at the Dept. of Oral and Maxillofacial Implantology of Shanghai Ninth People's Hospital will be screened and invited to participate while attending new patient clinics.

#### Eligibility Criteria

All potential participants will be assessed for eligibility based on the inclusion and exclusion criteria.

##### Inclusion Criteria

- Being edentulous or having a terminal dentition (22) and accepted treatment plan for fixed implant-supported prosthesis restoring at least 10 pairs of occluding teeth.
- Self-reported inadequate fresh vegetables or fresh fruits or protein foods intakes (daily intake thresholds based on the Chinese Dietary Guidelines for the Elderly recommendations).
- Understanding written and spoken Chinese and ability to respond to Chinese questionnaires.
- Able and willing to give informed consent for participation in the study.
- Able and willing to comply with 12-month follow-up.

##### Exclusion Criteria

- General and local contraindications to implant-supported immediate-loading fixed prosthesis.
- Looking for replacement of existing implant-retained overdenture with implant-supported fixed denture treatment.
- Presence of infectious disease, acute or chronic symptoms of TMJ disorder.
- Psychiatric disorder, dementia.
- Any dietary restriction, currently taking nutrient supplements or inability to choose his/her diet.
- Uncontrolled diabetes (HbA1c ≥ 7.0%).
- Self-reported heavy smokers (>10 cigarette/day).

#### Screening

Screening evaluations for this study will be performed in the context of routine patient evaluation in the clinic. The investigators will approach consecutive patients with the condition for possible inclusion in the study. During screening the investigator will also verify eligibility criteria. Additionally, social media (WeChat & Weibo) advertisement will be utilized to help recruit study participants. For some of these subjects, initial screening will be performed by phone. Following the telephone screening, the potential subjects will be invited for clinical screening evaluation.

#### Enrollment

Subjects fulfilling the inclusion and exclusion criteria will be invited to participate in the study and receive an explanation of the study, its objectives, benefits and risks by the investigator in the context of informed consent. The following information will be collected and recorded after the inclusion of the participants.

(1) Demographics: date of birth, gender, education, ethnicity, family income and anthropometric measures.
(2) Lifestyle factors: smoking (including tobacco consumption and smoking history), drinking habits and oral hygiene habits using the items from the Fourth National Oral Health Survey Questionnaire in Mainland of China (23) will be recorded.
(3) Health literacy: oral health literacy will be recorded using the Chinese version of the Short-Form Health Literacy in Dentistry (HeLD) scale (24) and nutrition literacy using the Nutrition Literacy Questionnaire for the Chinese Elderly (25).
(4) Medical History: Details of medical, including diabetes mellitus, cardiovascular diseases and other systemic diseases will be recorded. For subjects with diabetes, levels of glycated hemoglobin (HbA1c) will be obtained from the patients' medical records.
(5) Concomitant Medications: All over the counter or prescription medication, vitamins, and/or herbal supplements will be recorded on CRFs.

#### Randomization

Subjects will be randomly assigned to one of four groups with a 1:1:1:1 ratio by stratified block randomization. The block size will be 8 and stratifying factors will be diabetes status and smoking. Subjects will be registered into the study by a study registrar who will assign the treatment number and organize the sequence of the bookings of the patient according to the random allocation. The registrar will not be involved in any other study procedures.

#### Blinding and Allocation Concealment

Timing of treatment will be concealed to the therapists and to the examiners. Two separate masked therapists will perform the dental or the nutritional interventions. All laboratory assessments will be performed blindly.

#### Removal and Withdrawal Criteria

Those who have been selected for this trial and fall into one of the following circumstances are regarded as removed cases.

(1) Violation of important entry criteria;
(2) Receiving no study interventions;

Each participant has the right to withdraw from the study at any time. In addition, the investigators may discontinue a participant from the study at any time if the investigators consider it necessary for any reason including:

(1) Best interest of the patient.
(2) Ineligibility (either arising during the study or retrospective having been overlooked at screening).
(3) Significant protocol deviation.
(4) Significant non-compliance with treatment regimen or study requirements.
(5) An adverse event which requires discontinuation of the study or results in inability to continue to comply with study procedures.
(6) Inability to continue to comply with study procedures (e.g., moving to another city).
(7) Consent withdrawal.
(8) Loss to follow up.

If any subjects withdrawn from the study, no particular observation or treatment would need to continue. Subjects would be replaced if anyone withdrawn after the study started.

Regardless of the reason, complete clinical data should be retained for subjects who withdraw from the trial. The reason for withdrawal or early termination will be recorded in the CRF (case report form). If the participant is withdrawn due to an adverse event, if any, the investigator will arrange for follow-up visits until the adverse event has resolved or stabilized.

### Study Intervention

#### Treatment of Trial Participants

The treatment process will include provision of implant-supported full-arch fixed prostheses (dental intervention, DE) and dietary intervention tailored to the dental status (dietary intervention consisting of nutrition education, DI). Subjects will be randomized to either a waiting period or treatment regarding dental intervention and dietary intervention. Ethical justification for the waiting period comes from the current waiting list in the regular care at the hospital. In order to improve the compliance with the protocol, all subjects participating in the study will have an alert in their patient record identifying them as participants to this protocol to alert administrative staff on the need to follow a stringent timing of follow-up appointments.

#### Dental Intervention

All participants will receive implant-supported full-arch fixed prostheses in at least one jaw (26, 27) and appropriate treatment in the opposing jaw regarding periodontal disease, caries, replacement of missing teeth and soft tissue disorders to get at least 10 pairs of occluding teeth (22).

Before surgery, a treatment plan will be made according to the clinical examination, study model and the CBCT data. Pre-surgery mock-up will be produced to guide implant placement and to facilitate the fabrication of the immediate prosthesis. A surgical guide with tooth set-up will be used for implant placement and bite registration, as needed.

After administrating local anesthesia any remaining tooth will be exacted atraumatically and the sockets will be carefully curetted. A crestal incision will be made and a full thickness mucoperiosteal flap will be reflected. For the preparation of the osteotomy site for implant placement, a modification of the drilling protocol according to the manufacture's recommendation will be followed as needed for immediate placement in case of the presence of residual teeth/roots. Tapping may not be used depending on the bone density to ensure primary stability of the implant. After the site preparation, 4–8 Nobel Active® implants (Nobel Biocare, Goteborg, Sweden) will be placed. Multi-Unit abutments (Nobel Biocare, Goteborg, Sweden) will be placed onto the implants. The abutment will be tightened with a torque of 35Ncm for straight multi-unit abutments and 15 Ncm for angulated multi-unit abutments. Healing caps will be placed on the abutments to support the peri-implant mucosa. The flap will be closed with a 5-0 resorbable suture (Vicryl, Johnson & Johnson Medical, Pomezia, Italy). Then, splinted impression will be taken at abutment level using an individual open tray. Pre-surgery mock-up or surgery guide with tooth set-up will be used to register the occlusal relationship. Patients will receive amoxicillin (Xinya Co, 500 mg, 3 times/day for 7 days). Decongesting nasal drops (phenylephedrin, 0.1 ml, 3 times/day for 3 days) will be prescribed if sinus elevation will be performed. Mouth rinsing with chlorhexidine 0.12% three times per day and modified oral hygiene procedures will be prescribed for the first 2 weeks of healing (sutures still in place).

A screw-retained, metal-reinforced, acrylic resin interim restoration will be delivered within 24 h of surgery. All centric and lateral contacts will be assessed and modified, until occlusal contacts are uniformly distributed on the entire prosthetic arch. Sutures will be removed at 2 weeks. After a healing period of 4 month, a definitive screw-retained, full-arch prosthesis will be delivered.

#### Brief Nutrition Education

The nutrition education will be conducted based on the health Belief Model (HBM) (28) addressing perceived susceptibility and severity of lacking the targeted behavior, perceived benefits and barriers of carrying out the targeted behavior, cues to action, and self-efficacy. With behavioral goals being increasing an individual's likelihood of food intake regarding fresh vegetables, fresh fruits, and high-quality protein foods (i.e., poultry, meat and aquatic product), the nutrition education session has been designed to be culturally tailored.

Participants will receive a 20-min coordinated nutrition education in the form of a slideshow presentation by a nutritionist in the clinical setting. On completion they will receive a copy of a pamphlet prepared in three parts (overall dietary goal, recipe examples mainly composed of softer and easy-to-chew food, and recipe examples composed of various food without restriction on the texture). The advice has been compiled with reference to the 4th edition of Dietary Guidelines for Chinese Elderly Residents (2016) by the Chinese Nutrition Society (29) that will be given to the participant separately. If a participant does not prepare his or her own meals, the person who does the cooking receives the dietary advice as well. A dietary checklist aiming to evaluate the compliance will be delivered with the pamphlet and patients will send it back after 1-week's recording.

### Measurements and Outcomes

#### Timing of Assessment

Study assessments will be performed at baseline, 4, 8, and 12 months unless otherwise stated below. For Group B, C and D, an additional assessment will be performed at 16-month follow-up.

#### Food and Nutrient Intake

The primary outcome measure will be changes in intake of fresh fruits and fresh vegetables measured at 4 months using the 24-h dietary recall method. Protein% of total energy will also be calculated. Three 24-h dietary recall will be conducted through face-to-face interview, twice on weekdays and once on weekend. The data on food consumption will be converted into the corresponding nutrient contents based on the 6th version of China Food Composition Tables Standard Edition. Moreover, a modified simplified food-frequency questionnaire (FFQ) of 33 food group items (30) will be conducted at baseline, 4, 8, 12, and 16 months.

#### Masticatory Function

Masticatory function will be assessed at baseline (before treatment) and 4, 8, 12 and 16 months after insertion of a fixed implant retained prosthesis using the quantitative method described by Schimmel et al. (31) as previously described (32). In brief, subjects will be asked to mix a two-color chewing gum with 20 masticatory cycles. The obtained bolus will be pressed to a standardized height and a color image will be acquired. Quantitative data will be obtained by digital analysis of the image using variance of hue as the outcome.

#### Peri-Implant Soft and Hard Tissue Health

Peri-implant soft tissue condition will be measured by periodontal probing (UNC/CP-11.5B Screening Color-Coded Probe, Hu-Friedy, Chicago, IL, USA). Modified plaque index (mPI), probing depth (PD), and modified bleeding index (mBI) will be evaluated (33). Standardized panoramic radiographic imaging will be conducted to assess the peri-implant bone level. The assessment will be performed at 4-, 8-, and 12-month post-surgery.

#### Oral Health Impact Profile (OHIP)-14

The oral health impact profile (OHIP)-14 (34) will be administrated to assess the impact of oral health on the quality of life of participants using a validated Chinese translation of the instrument (35).

#### Biological Samples

Biological samples will be collected and processed in a standard way by dedicated study personnel blind with respect to treatment status. The blood sample collection will be scheduled at 8 a.m.-9 a.m. Patients will fast overnight (12–14 h) prior to blood collection. Subjects will be advised to avoid strenuous exercise 1 h prior to collection. Samples will be processed at the clinical research center laboratory to meet the preservation standards for the assay of each marker and will be either assayed immediately or stored at −80°C in the Shanghai Ninth People's Hospital Biobank facility for later analysis.

##### Metabolic and Inflammatory Biomarkers

The following biomarkers will be assessed by a specialized GCP approved clinical pathology laboratory.

a) Blood serum concentration of homocysteine.
b) Plasma hs-CRP, TNF-α, IL-1βand IL-6.
c) Co Q10, Uric acid and superoxide dismutase.
d) Blood lipids (total cholesterol, HDL cholesterol, LDL cholesterol, triglycerides and Lpa).

##### Plasma Nutrient Biomarkers

Nutrient biomarkers will be assessed at baseline, 4, 8, 12, and 16-month follow-up by an accredited laboratory according to international standards.

a) Plasma vitamins A, B2, B12, folate, C and E will be measured by liquid chromatography-mass spectrometry (LS-MS).
b) Plasma carotenoids (α- and β-carotene, β-cryptoxanthin, lycopene, lutein/zeaxanthin) and tocopherols (α- and γ) will be measured by LS-MS.

##### Plasma Metabolomics

a) Metabolites will be profiled with untargeted metabolomics using liquid chromatography coupled with mass spectrometry (36).
b) Oxylipin changes will be assessed by oxidative lipidomics (37).

##### Oral and Fecal Microbiome

Oral rinse, subgingival plaque and fresh stool samples will be collected for 16S rRNA gene sequencing at baseline, 4, 8, 12, and 16 months.

Oral microbiome samples will be obtained by oral rinsing for 1 min with 5 mL of buffer solution (38, 39). Additionally, in dentate patients a subgingival plaque sample will be taken from the deepest periodontal pocket/lesion with a sterile paper point inserted to the depth of the pocket. The subgingival plaque sample will be collected after isolating the sampling area with cotton rolls gentle air drying, and supragingival plaque removal. Samples will be immediately stored at −80°C.

Sterile stool tube with a spatula inside will be given to the participants with detailed instructions on how to collect the specimen. Fresh stool samples will be collected by the participants at home the night before the visit day or the morning of the visit day (40). Samples will be stored in the patient's refrigerator at 4°C until submission. During transportation, samples will be kept on ice in a cooling bag.

#### Nutritional Status

Mini-nutrient status form (MNA-SF) (41) will be used to screen patients for risk of malnutrition at baseline, 4, 8, 12, and 16 months.

#### Muscle Strength

A hand grip dynamometer will be used to assess muscle strength at baseline, 4, 8, 12 and 16 months essentially as described (32).

#### Cognitive Function

Cognitive function will be assessed with the Mini-Mental State Examination (MMSE) (42) and the Ascertain Dementia 8 (AD8) questionnaire (43) at baseline, 4, 8, 12, and 16 months.

#### Depression Symptoms

Depressive symptoms will be assessed with the shortened Center for Epidemiologic Studies Depression Scale (CES-D10) (44).

### Safety Evaluation

#### Adverse Events

The collective evidence from numerous clinical trials reveals consistent findings that the implant supported fixed full-arch prosthesis is a safe and effective treatment approach for terminal dentition or full edentulism. Dietary advice is also a safe intervention for edentulous elderly. No significant adverse events have been reported. Occasionally the patient may experience early implant failure and/or mechanical complications of the prosthesis. These will be recorded in the case report forms and will be managed according to standard of care with additional implant placement or refabrication/modification of the existing prosthesis.

#### Follow-Up for Adverse Events

Adverse events (AE), if any, will be managed according to current standard of care for the specific condition and will be reported to the ethics committee. The principal investigators will assess and manage the condition to the best of his knowledge and refer to specialist care if appropriate and in the best interest of the participant. AE will be considered resolved once the principal investigators concur that to be the case.

## Data Analysis

### Sample Size

The sample size has been determined based on the primary outcome: changes in the intake of fresh fruits and fresh vegetables. Based on the relevant studies where the average fruit and vegetable intake was about 255 ± 200 g per day in edentulous elderly (17), we assume that the average fruit and vegetable intake will increase 50 g, 70 g and 245 g in patients receiving dental prostheses alone, brief dietary advice alone, or the combination dental treatment with brief dietary advice, respectively. With an anticipated 20% loss to follow-up, 30 patients are needed in each group to test the significance of dental treatment, brief dietary advice, alone or in combination with alpha set at 0.05 and with 80% power. Due to limitations in the baseline knowledge for precise sample size calculations and the efforts required for an adequate pilot study, adaptive adjustment of sample size will be performed. Sample size calculation will be re-estimated after obtaining the primary outcome of 10 patients in each group. Based on the interim analysis for adaptive design, several scenarios have been identified: (i) the original sample size estimation is appropriate and the study will be completed accordingly; (ii) the original sample size will be insufficient but will still be within the capability of recruitment of the study center, in such case the sample size will be expanded according to the adaptive design principles; (iii) the original sample size will be insufficient but too large for successful completion of the study at the study center alone, in such scenario the study will be completed as a pilot study with the original sample size and a multicenter trial will be designed and implemented based on the results.

### Statistical Software and General Requirements

Data analysis will be performed in SPSS software, version 26.0 (IBM Corp., Armonk, NY, USA). The level of statistical significance will be set at 0.05 for all tests.

### Statistical Analysis Plan

(1) Descriptive statistics will report the demographic, clinical and biological characteristics of the study population. Means with standard deviations (SD) or medians with interquartile range (IQR) will be used to describe continuous variables. Frequencies will be used to describe categorical variables.
(2) The normality of clinical and biological parameters at baseline and each re-evaluation visit will be tested for normality using the Kolmogorov-Smirnov test. The homogeneity of the clinical and biological parameters at baseline and each re-evaluation visit will be tested using Levene's test.
(3) ANOVA analysis will be used to test the effect of dental treatment, brief dietary advice, both dental treatment and brief dietary advice, sequence of two interventions for continuous variables with a normal distribution. The Kruskal–Wallis test will be used for non-continuous variables or continuous variables not normally distributed.

### Data Quality and Assurance

All investigators involved in this study will be trained appropriately for the standard operating procedures including the questionnaire conduction, blood sampling and preservation, presurgical examinations and treatment (regarding periodontal diseases, caries, missing teeth and soft tissue disorders) and delivery of interventions.

(1) The 24-h dietary recall will be conducted by clinical nutrition specialists trained appropriately for the standardized interviewing procedures and assessment of dietary intake. The inter- and intra-examiner reliability with respect to the measurement of fresh foods and fresh vegetables intake will be assessed by the intraclass correlation coefficient (ICC). In order to ensure optimal inter- and intra-examiner reliability, ICCs needs to be more than 0.75.
(2) The therapists, who will deliver the dental intervention, will be experienced specialists in implant dentistry fulfilling the Shanghai requirements. Treatment will be provided to the satisfaction of the clinician and patient. For logistic reasons, five therapists will be included in this study.
(3) The investigator who performs dietary interventions will be trained in delivering a standardized dietary instruction session including the verbal instruction and demonstration of the pamphlet. In addition, the investigator will be trained in the use of questionnaires.

Regular monitoring for assuring protocol compliance, and data quality at the clinical site, including review of source documents and records, consent forms, etc will be performed by an investigator trained in both GCP and the specific procedures. Furthermore, the clinical research coordinator will audit the case report forms for the first few patients to ensure correct filling of forms. The study will also be monitored by the compliance office of the National Clinical Research Center of Oral Diseases and Clinical Research Center of the 9th People's Hospital.

### Confidentiality

All trial-related data will be stored securely at Shanghai PerioImplant Innovation Center. The participant information will be stored in locked cabinets with limited access. All data will be anonymized by assigning a Research ID used for data collection and processing to maintain participant confidentiality. All records containing personal identifiers will be stored separately from study records identified by the research ID number.

## Discussion

Many older adults with severe tooth loss and masticatory dysfunction change their food choices and incorporate softer food with more carbohydrates and fats and depleted of essential micronutrients and fibers. They also progressively lose weight, become frail and dependent on others for their daily necessities. Replacement of missing teeth alone restores masticatory function but does not positively influence diet. Great attention is currently being paid to the combination of dental and dietary interventions. Their relative importance and optimal sequence, however, remain unknown. This lack of knowledge has far-reaching consequences in the design of optimal treatment regimens and testing their health benefits in definitive studies. The present study will provide critical information with major implications for caring of individual subjects and for public health and policy (45).

The design of this trial has posed significant challenges in terms of experimental design, choice of the population/condition, definition of the dental and the dietary interventions as well as the choice of outcomes. These will be briefly discussed following the PICOT format.

The selected 2 × 2 factorial randomized clinical trial design provides greater efficiency in terms of sample size while allowing testing of multiple clinically relevant questions on the relative effect size of dental and/or dietary interventions. The incorporation of an adaptive design that will recalculate sample size after data will be available from a third of the planned subjects provides robustness to the approach even considering the possible imprecision of the preliminary data used for sample size calculation. While sample size assumptions have been piloted and confirmed in the specific patient population the approach offers added robustness against type II errors. This is particularly important given the high costs of rendering the treatment to this population and the consequent difficulty in properly funding a pilot trial. Specific a priori scenarios have been identified with regards to completion of the trial.

Tooth loss is frequently incremental over the course of life and subjects in the population present with a spectrum of severity of loss of masticatory function. This study will focus on the more severe end of the spectrum as these subjects are both likely to suffer from greater changes in diet and more likely to show improvements in masticatory function because of tooth replacement. It will also recruit aging subjects who represent most edentulous subjects. Additional studies expanding the observations to milder forms of edentulism will be needed. To ensure that subjects will suffer from both masticatory dysfunction and a degree of malnutrition, an inclusion criterion has been added in terms of verification of poor fruit, vegetable, or protein intake. Pilot nutritional analysis of edentulous subjects reporting for treatment in the specific setting has verified that most of them reported at least one aspect of impairment and fit the inclusion criteria. These aspects are important for the external applicability of the results of the trial and ongoing epidemiologic research will provide additional information.

The definition of both the dental intervention and the nutrition education are also notable. To address masticatory dysfunction this study will employ fixed dentures supported by dental implants—a well-defined intervention routinely performed in the specific setting—as these have been shown to provide better objective and subjective chewing benefits (11). The masticatory function will be restored to provide at least 10 occluding pairs of teeth, a number generally considered compatible with adequate function (22, 46).

HBM has been used in aiding behavior change intervention for decades, and it has been applied to Asian populations. With the HBM-based nutrition education, the objective is to motivate participants from the pre-contemplative stage to the contemplate stage, and even to preparatory stage with the materials provided. Combining with the dental intervention which will solve the physical barrier, the hypothesis is that participants will progress to the executive stage at home. During the follow-up period, the importance of dietary intake will be reinforced to help them to stay in the maintenance stage. The intervention, its instruments and their delivery have been tailored to local circumstances, evaluated, and revised in our pilot study before implemented for the trial. Details are presented in the online appendices as a potential resource for additional trials.

While the equipoise to justify randomization is strong, recruiting patients with edentulism/terminal dentition for a trial is challenging due to the severity of the condition and the impact on quality of life. The opportunity arises in the specific setting due to the waiting list for treatment that justifies the delay in the delivery of the care initially sought by the patient.

Lastly the choice of the primary outcome has been complex due to the limited previous information on clinically relevant outcomes and the need to maintain the size of this trial within the recruitment possibilities of the single center. The choice to focus on a proxy outcome—changes in fresh fruits and fresh vegetables consumption—as the primary outcome, rather than a health gain measure, is based on the need to establish the effectiveness of the treatment regimen and logistic considerations. The limitation of 24-h recall method in providing an accurate estimate of long-term energy intake has been realized. Thus, the study plans to combine food frequency questionnaires which replied on generic rather than specific memory to offer detailed assessment of the study period. Furthermore, the study plans to assess a wide palette of secondary outcome that will provide insight into mechanisms of a potential benefit by exploring both biochemical markers, metabolomics and changes in the oral-gut microbiome axis and functional quality of life instruments.

The relatively short follow-up time for the factorial design component of the study is adequate to assess the efficacy of the interventions. The 12-month extension is relevant as it will supply critical information about retention of subjects in the trial and medium-term compliance with the dietary intervention and effectiveness of the dental intervention. It will also provide the basis for future longer-term trials focusing on health outcomes.

## Ethics Statement

The studies involving human participants were reviewed and approved by the Institutional Review Board of the Shanghai Ninth People's Hospital, Shanghai Jiao Tong University School of Medicine (Approval No. SH9H-2021-T321-3). The patients/participants provided their written informed consent to participate in this study.

## Author Contributions

MT conceived and designed the study. CY, H-CL, S-JQ, and J-YS contributed to the study design and protocol development. H-CL, BL, JS, S-CQ, YT, and KD assisted with preliminary analyses on the patient population, piloting of material, and preparation of study launch. XZ provided the sample size calculations and the statistical plan. S-JQ, JS, and BL drafted the manuscript based on the original protocol. All authors revised and approved the final version of the manuscript.

## Funding

This study has been supported by the Project of Biobank from Shanghai Ninth People's Hospital, Shanghai Jiao Tong University School of Medicine (YBKB202102 and YBKA201906).

## Conflict of Interest

The authors declare that the research was conducted in the absence of any commercial or financial relationships that could be construed as a potential conflict of interest.

## Publisher's Note

All claims expressed in this article are solely those of the authors and do not necessarily represent those of their affiliated organizations, or those of the publisher, the editors and the reviewers. Any product that may be evaluated in this article, or claim that may be made by its manufacturer, is not guaranteed or endorsed by the publisher.

## Abbreviations

DE: dental intervention
DI: dietary intervention
HbA1c: glycated hemoglobin
CRF: case report form
CBCT: Cone beam computed tomography
hs-CRP: high-sensitivity-C-reactive protein
TNF-α: tumor necrosis factor-α
IL-1β: Interleukin-1β
IL-6: Interleukin-6
Co Q10: coenzyme Q10
LS-MS: liquid chromatography-mass spectrometry
HDL: high density lipoprotein
LDL: low density lipoprotein
HBM: health belief model.
