# Supplementary material for: Effects of Dental Implants and Nutrition on Elderly Edentulous Subjects: Protocol for a Factorial Randomized Clinical Trial
Source: Front Nutr. 2022 Jun 27;9:930023. doi: 10.3389/fnut.2022.930023 (PMC9272417; doi:10.3389/fnut.2022.930023)
Supplement: Supplementary file 1 [file Data_Sheet_1.docx]

**Appendix 1.**

**Calculation method of self-reported vegetable or fruit or protein intakes as one of the inclusion criteria**

Patients were included in the study if at least one of the three criteria listed below were met (Patients were excluded if none of three criteria was met).

I. Patients with an average daily total intake of < 80 g of poultry, meat and aquatic product.

II. Patients with an average daily intake of < 200 g of fresh fruits.

III. Patients with an average daily intake of < 300 g of fresh vegetables.

**Part I：Record sheet and calculation method**

1. Please recall your usual eating habits. Regarding the intake of poultry, meat and aquatic products (chicken, duck, pork, beef, lamb, fish, shrimp), do you eat them more than three days a week?
   1. No-> 0 points
   2. be

If yes, does the average daily intake reach __80__g

1. No-> 0 points
2. Yes-> 1 point
3. Please recall your usual eating habits. Regarding the intake of fresh fruits, do you eat them more than three days a week?
   1. No-> 0 points
   2. be

If yes, does the average daily intake reach __200__g

1. No-> 0 points
2. Yes-> 1 point
3. Please recall your usual eating habits. Regarding the intake of fresh vegetables, do you eat them more than three days a week?
   1. No-> 0 points
   2. be

If yes, does the average daily intake reach __300__g

1. No-> 0 points
2. Yes-> 1 point

Final calculation of total score (out of 3)

🡪If the patient scored less than 3, then he/she was included.

🡪If the patient scores 3, then the patient was excluded.

**Part II：Reference materials for deciding the threshold.**

The threshold used for the inclusion criteria is based on the 4th edition of Dietary Guidelines for Chinese Elderly Residents (2016) by the Chinese Nutrition Society. Since patients with terminal dentition or total edentulism are generally characterized as normal to underweight, the above threshold is recommended based on a population at 1600kcal energy level.

**Balanced dietary patterns and recommended food amounts (g/day) for the different energy needs of the elderly**

| **Energy level** | **1400kcal** | **1600kcal** | **1800kcal** | **2000kcal** | **2200kcal** | **2400kcal** |
| --- | --- | --- | --- | --- | --- | --- |
| **vegetables** | 300 | 300 | 400 | 450 | 450 | 500 |
| **fruit** | 150 | 200 | 200 | 300 | 300 | 350 |
| **Aquatic products** | 40 | 40 | 50 | 50 | 75 | 75 |
| **Livestock meat** | 40 | 40 | 50 | 50 | 75 | 75 |
| **egg** | 25 | 40 | 40 | 50 | 50 | 50 |
| **soybeans** | 15 | 15 | 15 | 15 | 25 | 25 |

Adapted from ‘Dietary Guidelines for Chinese Elderly Residents’ by Chinese Nutrition Society, 2016 [M], Beijing: People's Health Publishing House
