# Supplementary material for: Effects of Dental Implants and Nutrition on Elderly Edentulous Subjects: Protocol for a Factorial Randomized Clinical Trial
Source: Front Nutr. 2022 Jun 27;9:930023. doi: 10.3389/fnut.2022.930023 (PMC9272417; doi:10.3389/fnut.2022.930023)
Supplement: Supplementary file 2 [file Data_Sheet_2.docx]

**Supplementary 2.**

**Health Belief Model variable summary and potential intervention strategies**

The potential intervention strategies were illustrated by focusing on the following six aspects.


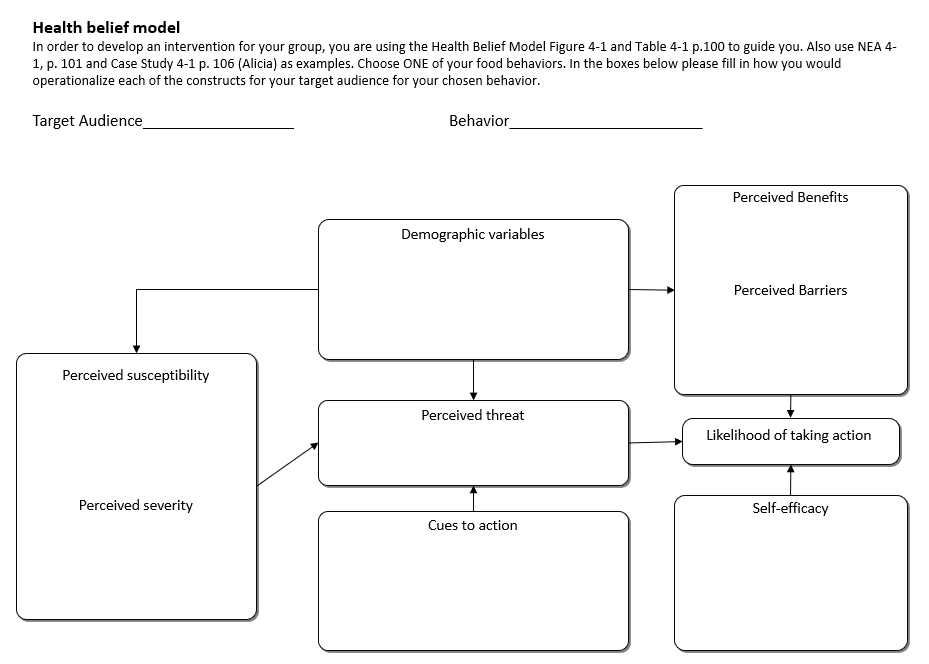


Adapted from ‘Nutrition education: linking research, theory, and practice’ by Contento, Isobel R, 2007.

**1 Perceived Threat**

**1.1 Perceived susceptibility**

- Help patients develop accurate perceptions of their dental status and their eating habits in the past few years, such as insufficient intake of vegetables, fruits, and high protein foods. Such compromised nutrition intake may lead to an increased risk of malnutrition.
- Help them to realize that the way to prepare extreme soft and easy-to-chew food could destroy the nutrients in the food, thus imposing a higher risk of nutrient deficiency than ordinary cooking methods.
  1. **Perceived severity**

Help individuals develop realistic perceptions of the consequences of compromised nutrition intake. Individuals with imbalanced diet are more likely to have higher nutritional risks, which might lead to malnutrition, underweight and sarcopenia.

- Insufficient intake of fruits and vegetables may increase the risk of cancer and chronic metabolic diseases.
- The deficiency of high-quality protein may increase the susceptibility to non-communicable diseases; undermine physical and cognitive functions and fast aging process among older adults.

**2 Behavioural Evaluation**

**2.1 Perceived benefits**

- Fruits, vegetables, and protein contribute to a healthy and balanced diet, which can provide comprehensive nutrients, facilitate wound healing and promote long-term health.
- Adequate protein intake can help to reduce the risk of sarcopenia, osteoporosis, and frailty.
- Vegetables and fruits, which are rich in antioxidants and vitamins, can help reduce the risk of heart disease, stroke and some cancer.
- More opportunities to eat and interact with family members.

**2.2 Perceived barriers**

- Understand how to prepare the food in the right way
- The knowledge of recommended intake frequency and amount of food.

**3 Cues to action**

- Set daily goal on dietary intake and daily checklist as an external reminder.
- Monitor weight fluctuations weekly.

**4 Self-efficacy**

- With the information from dietary advice, patients with terminal dentition or complete edentulism are capable of improving cooking methods and preparing softer vegetables, fruits and protein.
- Patients who have received dental rehabilitation are capable of eating diverse kinds of food.
- If needed, patients’ families can also help cook meals in accordance with the dietary guidelines.
